# Nanoscale Imaging of Caveolin-1 Membrane Domains *In Vivo*

**DOI:** 10.1371/journal.pone.0117225

**Published:** 2015-02-03

**Authors:** Kristin A. Gabor, Dahan Kim, Carol H. Kim, Samuel T. Hess

**Affiliations:** 1 Department of Physics and Astronomy, University of Maine, Orono, Maine, United States of America; 2 Graduate School of Biomedical Sciences, University of Maine, Orono, Maine, United States of America; 3 Department of Molecular and Biomedical Sciences, University of Maine, Orono, Maine, United States of America; Cornell University, UNITED STATES

## Abstract

Light microscopy enables noninvasive imaging of fluorescent species in biological specimens, but resolution is generally limited by diffraction to ~200–250 nm. Many biological processes occur on smaller length scales, highlighting the importance of techniques that can image below the diffraction limit and provide valuable single-molecule information. In recent years, imaging techniques have been developed which can achieve resolution below the diffraction limit. Utilizing one such technique, fluorescence photoactivation localization microscopy (FPALM), we demonstrated its ability to construct super-resolution images from single molecules in a living zebrafish embryo, expanding the realm of previous super-resolution imaging to a living vertebrate organism. We imaged caveolin-1 *in vivo*, in living zebrafish embryos. Our results demonstrate the successful image acquisition of super-resolution images in a living vertebrate organism, opening several opportunities to answer more dynamic biological questions *in vivo* at the previously inaccessible nanoscale.

## Introduction

Many crucial biological processes occur on length scales that are inaccessible to conventional light microscopy techniques. This highlights the importance of techniques that can image at high resolution and provide valuable nanoscale information from single molecules. Conventional fluorescence microscopy with labeling techniques has brought revolutionary advances in our understanding of protein distributions and functions in the past two decades, albeit at diffraction-limited resolutions. The technique described herein, *in vivo* FPALM, enables construction of super-resolution images from localizations of individual molecules in a living zebrafish. Such studies are likely to lead to a plethora of opportunities for studies relating to dynamic biological processes such as the pathogenesis of various diseases, the immune response to pathogen invasion of the host and real-time movements and interactions of proteins of interest.

In recent years, techniques have been developed which can achieve super-resolution using localization of large numbers of optically resolvable single molecules [1–3] or stimulated emission depletion [4], achieving effective resolutions in the range of 20–30 nm. Localization-based super-resolution microscopy methods have been shown to image living cells [5], three-dimensional specimens [6,7], and multiple fluorescent species [8–10]. These methods, however, do not provide super-resolution single molecule information in a living organism. Here, fluorescence photoactivation localization microscopy (FPALM) imaging in a living zebrafish embryo is demonstrated using widefield illumination, enabling imaging of single molecules in a thick sample with an effective resolution of ~ 45 nm.

Previous super-resolution microscopy studies utilizing living species [5,11–13] have not been performed in a living vertebrate organism. Despite the invaluable information that *in vitro* studies can offer, the molecular membrane organization observed in such systems may not fully reflect intact processes that occur in functioning and interacting tissues of a living organism. Studying the dynamics of individual Cav-1a molecules in a living zebrafish embryo can elucidate processes that take place at caveolae during the course of embryonic development. Cav-1a was selected based on its distinct size, morphology, and estimated number of molecules in a given caveolae. Previous *in vitro* studies using zebrafish cells revealed that Cav-1 was critical for antiviral signaling because when Cav-1 was depleted, clusters of Cav-1 molecules were dispersed, resulting in an abrogated immune response to virus infection [14]. These studies helped inspire the development of this technique for *in vivo* imaging. Using FPALM, we imaged Dendra2 [15,16] genetically fused to the zebrafish Caveolin-1a (Cav-1a) membrane protein, which has been shown to serve as the primary protein responsible for the formation of caveolae membrane domains [17,18]. An FPALM setup with widefield illumination was used to visualize individual molecules in cells of living zebrafish embryos. Our results demonstrate the presence of caveolae membrane domains in a living organism, consistent with previous studies that we have performed using zebrafish cells [14]. Applications of *in vivo* FPALM techniques provide opportunities to ask and answer a multitude of questions in a living organism at diffraction-unlimited nanoscales. In these studies, we used an exposure time of ~3 ms per frame and achieved a localization precision of ~ 40 nm and density of ~9500 molecules/μm^2^ with a total of 10–15 seconds of acquisition time per rendered image (3000–5000 frames per image).

To perform measurements in a physiologically relevant system, we have extended FPALM to the level of a living vertebrate organism. Such studies enable validation of previous findings in an *in vivo* model system. The zebrafish, *Danio rerio*, was used as a model organism for this study. Zebrafish embryos are an ideal model organism for *in vivo* microscopy studies due to their optical clarity, size, and amenability to genetic manipulation. For instance, zebrafish have been used for real-time imaging of GFP-labeled cells, or fluorescently labeled proteins or pathogens being expressed in a living embryo. In addition, use of the zebrafish *casper* mutant [19] which was genetically modified to be transparent for the lifetime of the fish, afforded low background levels in the present study. Using the zebrafish, we demonstrate that it is possible to perform FPALM in a living vertebrate by imaging cav1a-dendra2 *in vivo* in living zebrafish embryos. Our results demonstrate the successful image acquisition of super-resolution images in a living vertebrate organism and present new opportunities to answer more dynamic biological questions in functioning tissues of a living organism.

## Materials and Methods

### Ethics Statement

Zebrafish used in this study were handled in accordance with the recommendations in the Guide for the Care and Use of Laboratory Animals of the National Institutes of Health. The protocol was approved by the Institutional Animal Care and Use Committee (IACUC) at the University of Maine. IACUC approved guidelines for zebrafish care followed the standard procedures (www.zfin.org) of a 14 h light, 10 h dark cycle at 28°C.

### Zebrafish Care and Maintenance

Zebrafish were maintained in the Zebrafish Facility at the University of Maine, Orono. The facility was maintained according to IACUC standards. Embryos were obtained by natural crosses. Fertilized eggs were collected and raised in egg water (60 μg/mL Instant Ocean sea salts (Aquarium Systems, Mentor, OH)) at 28°C. The zebrafish used for these studies were either wild-type AB or *casper fms* embryos [19], which is a mutant line in which the embryos display no pigmentation. Studies were also performed in age-matched wild-type embryos and similar results were achieved.

### Microinjection of DNA into Zebrafish

Cav1a-HL4-dendra2 DNA was linearized by restriction enzyme digestion. The resulting DNA was purified using the PCR Purification Kit (Qiagen, Venlo, Netherlands) and quantitated by Nanodrop spectroscopy. Purified DNA (100 pg/embryo) was microinjected into the cell of zebrafish embryos at the one-cell stage. For some experiments DNA was co-injected with morpholino oligonucleotide (MO) targeted against Cav-1a as previously described [14]. Microinjection was controlled by a MPPI-2 pressure microinjector (Applied Scientific Instruments) and pulled microcapillary pipettes (Sutter Instruments, Novato, CA) to inject the plasmid. Injected embryos were then allowed to develop in egg water at 28°C. Prior to imaging, embryos were manually dechorionated.

### Super Resolution Imaging with FPALM

A 1-day-old zebrafish embryo (between 24 and 30 h post fertilization (hpf)) was anesthetized in a non-lethal dose of tricaine (4 mg/ml) solubilized in egg water. The fish was place on its side on a depression microscope slide in a drop of tricaine. A #1.5 glass coverslip was placed over the depression before being mounted on an FPALM setup that has been previously described [2,5,20]. Briefly, the setup constitutes an inverted microscope (IX71, Olympus America, Melville, NY) with a 60X water immersion objective (NA 1.2, UPLAPO60XW, Olympus America, Melville, NY). A 405 nm diode laser (BCL-405–15, Crystalaser, Reno, NV) was used to activate photoswitchable molecules in the sample. A motorized filter wheel (FW102, Thorlabs, Newton, NJ) containing neutral density filters provided manual incrementing of the activation laser intensity in steps of ~10^0.5^ (or 3.16-fold) to maintain a density of resolvable molecules of ~ 1 μm^2^. A 556 nm diode laser (LRS-556-NM-100–10, Laserglow, Toronto, Canada) with an intensity of ~15,000 W/cm^2^ at the sample was used to readout active molecules. Both beams were focused approximately at the back aperture of the objective lens to produce widefield illumination at the sample. Fluorescence from the sample was collected by the same objective, and filtered with a T565LP dichroic mirror (Chroma Technology, Rockingham, VT) and bandpass filtered with an ET605/70M filter (Chroma). The image was detected by an electron multiplying charge-coupled device (EMCCD) camera (iXon+ DU897DCS-BV, Andor Scientific, South Windsor, CT) operated at an EM gain of 200, with an exposure time per frame of 3 ms and a total acquisition time of ~10 s. Additional lenses (f = +60mm and f = +200 mm, Newport Corporation, Irvine, CA) were arranged as a telescope in the detection path to provide additional magnification, before the image is projected onto the camera CCD. A 256x145 pixel region of interest was defined on the camera, with an effective pixel size of 138 nm at the sample, which is determined by measuring how many camera pixels span across 100 μm distance of a scale placed at the sample. The camera was controlled using Solis software (Andor). Images were acquired using LabVIEWsoftware (National Instruments Corporation, Austin, TX). Note that while at present there is no field-specific standard, the super-resolution microscopy data obtained here (including rendered images and localized molecular coordinates), will be made available upon request.

**FPALM Analysis.** FPALM analysis was performed as previously described, using custom software written in MATLAB (Mathworks, Inc. Natick, MA). Raw frames (1000–5000 frames) were background subtracted using the rolling ball method [21] prior to the standard localization routine. Positive intensity peaks with at least one pixel above a minimum threshold were fitted to a two-dimensional Gaussian to determine the x and y coordinate, amplitude, 1/e^2^ radius, and offset of each point spread function (PSF). During the localization procedure, one of two positive intensity peaks within 621 nm (corresponding to 4.5 pixels) of each other was discarded to ensure localization of only one PSF in the diffraction-limited region. Further, when localizations occur within 300 nm in two or more consecutive frames, only one of the localization was retained in order to prevent multiple counting of the same PSF.

Clusters were identified as described previously [22] using a single-linkage cluster analysis. Briefly, clusters were determined based on molecular positions identified during the localization analysis. The positions of molecules that were within a maximum distance of 30 nm were defined as being within the same cluster. All molecules within 30 nm of a given molecule were identified using an iterative approach and then new molecules were added for neighbors within 30 nm. This process was repeated until all members of a given cluster were obtained. The number of molecules per unit area (density) was determined based on the coordinates of molecules determined to lie within the same cluster. The density of clusters was calculated as the number of molecules per square micron of the area in the cluster.

Sample drift was corrected using methods similar to those published previously [7,23,24]. All final images shown were drift corrected. Drift was corrected by calculating cross-correlation between transmitted light images taken before and after FPALM acquisition using the same EMCCD camera used for FPALM image acquisition. The displacement of the sample was calculated by determining the cross-correlation between the two images using the pixel counts of the transmitted light images and fitting the central peak of the resulting cross-correlation surface with a 2D Gaussian. The trajectory of the drift was linearly interpolated between the two end points of the displacement, and the localized molecule positions were then subtracted by the amount of drift interpolated according to their frame number. In cases where the cross-correlation peak was not strong, the particular data set was discarded to ensure proper compensation of a sample drift. All analysis was performed using custom software in MATLAB (Mathworks, Natick, MA).

## Results

### Schematic of Sample Preparation

**Figure 1:**
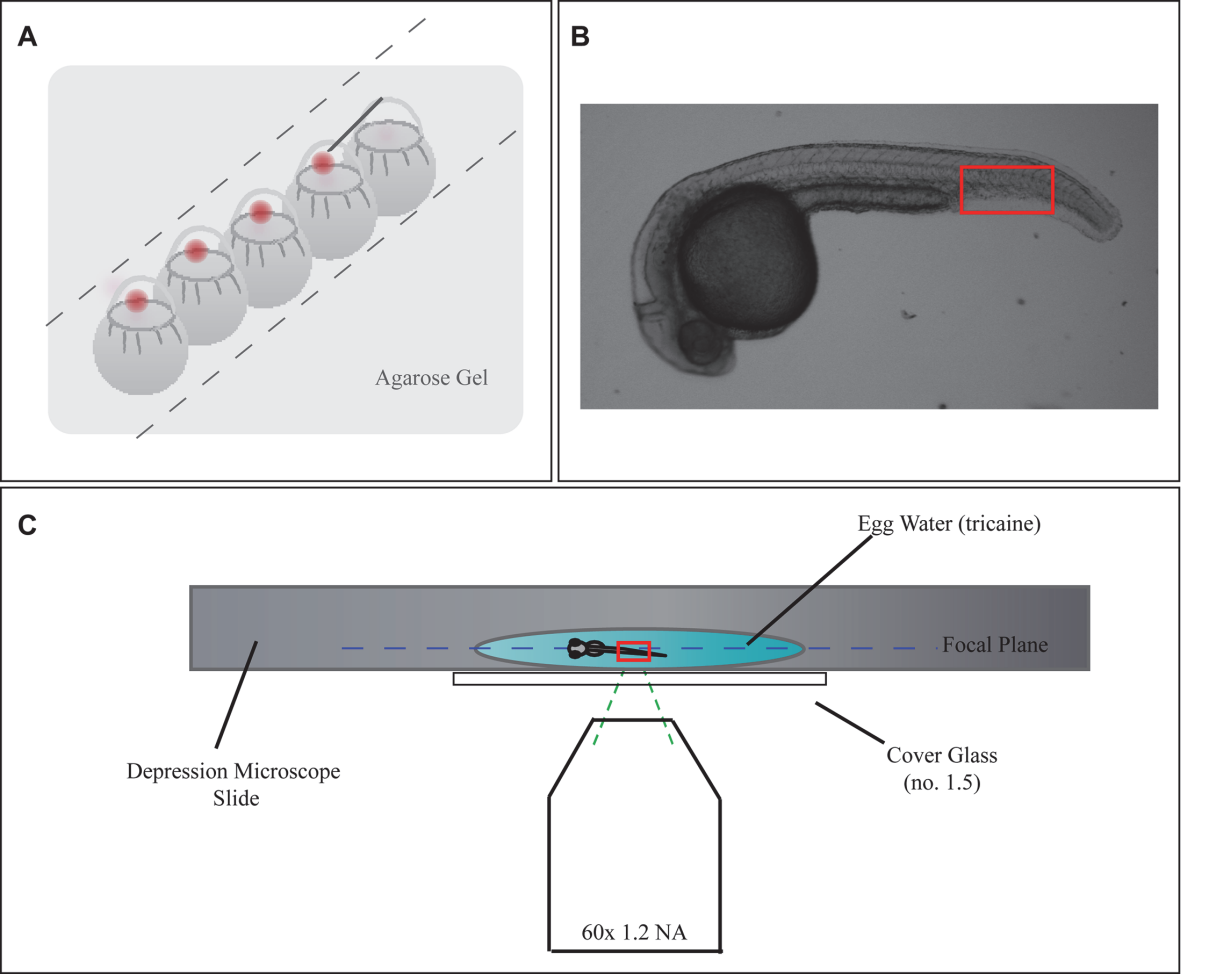
Widefield FPALM Enables Penetration Into Sample. Schematic of experimental setup. **A)** Diagram demonstrating how *Casper fms* embryos were injected at the 1-cell stage with linearized DNA plus phenol red indicator. **B)** A widefield image of a 24 hpf zebrafish embryo. Outlined in red is the region of the tail that FPALM images were acquired at, as it is the thinnest part of the fish and also has minimum autofluorescence. **C)** A 24 hpf embryo was placed in a microscope slide with a single shallow depression, immersed in a drop of non-lethal tricaine (solubilized in egg water). A cover slide (#1.5) was placed over the slide, covering the embryo and the coverslip was mounted on a microscopy setup suitable for FPALM.

FPALM was used to image a living zebrafish embryo (Fig. 1) as briefly described in materials and methods. Embryos that had been injected at the 1-cell stage with linearized plasmid (with phenol red indicator) were manually dechorionated at 24 h post injection prior to imaging. Since fish were injected immediately after fertilization, this corresponds with the 24 hpf developmental stage. It should be noted that the phenol red indicator would have dissipated by the time imaging was performed and was not a contributing factor to background fluorescence. For all images, a region of the fish was selected for imaging by initial viewing of unconverted Dendra2 through the microscope using transmitted light.

### Identification of Single Molecules *in vivo*

Using the FPALM setup described in Materials and Methods, images on the computer preview screen, prior to acquisition, were visually examined for single molecule fluorescence emission with diffraction-limited profile consistent in size and duration with the PA-FPs being activated and excited. At the above pixel size and laser intensities we employed for the PA-FPs used in our studies, the PSF of molecules were observed to have a diameter of 2–3 pixels and were visible for 1–4 frames, or ~3–12 ms in duration. Following this confirmation of well-profiled PSFs, images were acquired as described in Materials and Methods. Note that the images and analysis applies only to the localized, fluorescent Cav-1a molecules and does not account for the endogenous, non-fluorescent Cav-1a molecules that may be present in the analyzed cells.

**Figure 2:**
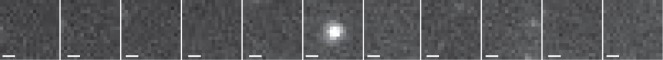
Identification of Single Molecules *in vivo*. The image demonstrates the ability to identify and localize single molecules above background in a living zebrafish embryo. Each image shows *a single frame during image acquisition* of a cell in an embryo during localization analysis. *Casper fms* zebrafish were injected at the 1-cell stage with plasmid DNA and images were captures 24 h post injection (i.e. 24 hpf). A single molecule is shown over consecutive frames, where the molecule is not present, turned on during the frame in which it is activated and localized, and then presumably has photobleached during acquisition of the subsequent frames. Note that the single molecule image appears to be approximately the width expected from the diffraction limit. Brightness and contrast were linearly adjusted in ImageJ for all frames for presentation purposes. *Scale bar*, *500 nm*.

For the localization analysis of acquired frames, single photoactivated molecules were identified and localized as described previously [2,10,20,25,26]. Some photoactivatable fluorescent proteins are known to reversibly photoswitch. However, dendra2 was in part selected because it demonstrates in irreversible photoconversion [15] and has limited susceptibility to reversible switching [27]. Typical background noise ranged from ~7–12 photons. Similar background levels were observed in control embryos injected with DNA elution buffer (data not shown). Further, to demonstrate the cycle of activation, localization, and photobleaching, analysis was performed on consecutive raw frames of data taken from an image of an embryo that was injected with pcDNA3.1 cav1a-HL4 dendra2 plasmid. A single raw frame which has an activated molecule that was identified and localized during analysis is shown (Fig. 2). The five raw frames prior to the molecule are shown as well as the five raw frames subsequent to the activated molecule. This sequence of raw frames shows no molecule, an activated molecule which was localized, followed by no visible molecule, presumably due to photobleaching.

Typical super resolution techniques such as FPALM offer localization precisions of ~20 nm laterally, which is ~10-fold greater than resolutions of conventional optical microscopies. However, due to their fine resolution, such techniques are more sensitive to sample drift, which could lead to blurred images with poor resolution or misinterpretation of data, especially in an anesthetized but living organism. We applied our *in vivo* images with methods which correct for two-dimensional drift in a manner that has been previously reported [1,7,23,24]. This correction is performed using transmitted light images without extraneous fiduciary markers [1,3,28].

During image acquisition, sample or instrument drift can occur over the range of several hundred nanometers. For super-resolution imaging techniques, even a small drift can distort images, depending on the size of the structure in question [1,7,23,24]. In the past, fiduciary markers such as gold nanoparticles, quantum dots, or fluorescent beads have been introduced into the sample to aid with drift correction. However, these methods require the addition of markers into a sample, potentially perturbing the biology itself, and the instrument may need to be adjusted to image or accommodate such markers. An alternative to fiduciary markers relies on the structure itself to be used to measure and compensate for drift [7,24]. In applications of our in vivo imaging, the dynamic rearrangement of structures may be the question of interest, and such methods cannot be directly used here. As an alternative, we applied the cross-correlation of transmitted light images, before and after the acquisition, which does not change as rapidly as cellular membrane constitutions, under suitable conditions. For our studies, it was assumed that drift within a single recorded camera frame was negligible. We excluded data sets for which we could not identify a clear peak in the cross-correlation between the transmitted light images, taken before and after the FPALM acquisitions. A lack of such cross-correlation peak indicates great morphological changes of the cell during the imaging, which would interfere with the ability to reliably determine the sample drift using this method. Uncertainty of our drift-correction was determined by calculating 95% confidence intervals for the Gaussian fitting of the cross-correlation peaks. For the drift-correction data set (n = 7 cells), the uncertainty of our drift correction ranged from 7–37nm, all below the localization precision of 40nm in our work.

### *In vivo* visualization of Caveolae-Like Membrane Domains Using FPALM

**Figure 3:**
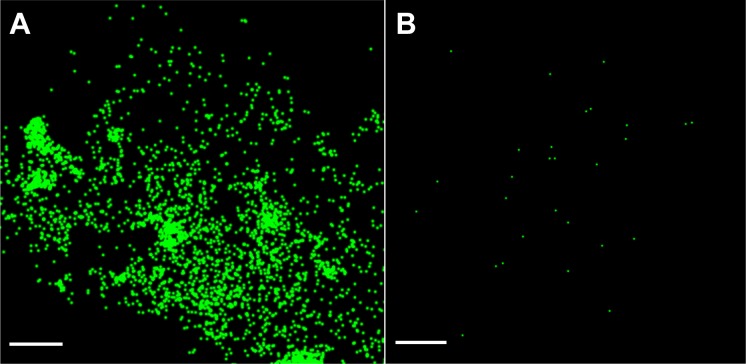
Cav-1 Specificity of Localized Molecules Demonstrates *In vivo* Visualization of Caveolae. *Casper fms* zebrafish were injected at the 1-cell stage with plasmid DNA and images were captured 24 h post injection (i.e. 24 hpf). A) Control MO + Cav-1b dendra2 demonstrates single cav1 molecules are localized. B) Image rendered from Cav1b MO+Cav-1b dendra2 embryos with knockdown of Cav1b expression demonstrates that the molecules seen in control are in fact Cav1b molecules. *Scale bar*, *1 μm*.

To test the specificity of the pcDNA3.1 cav1a-HL4 dendra2 images, a Cav-1a MO was used to knockdown the expression of Cav-1a protein. Zebrafish embryos were injected at the 1-cell stage with both plasmid DNA (cav1a-Dendra2, Fig. 3*A*) or Cav-1a MO (Fig. 3*B*). To confirm that no effects were a result of the MO injection, Control MO was also co-injected with cav1a-HL4-dendra2, and no differences were seen as a result of the MO (data not shown). Prior to imaging, embryos were manually dechorionated (24 h post injection) before being immersed in tricaine for imaging. Caveolae were observed in embryos injected with cav1a-Dendra2 (i.e. no Cav-1a MO knockdown) (Fig. 3*A*). However, in embryos injected with Cav-1a MO and cav1a-Dendra2, very few (29) molecules were localized (Fig. 3*B*), demonstrating that upon knockdown of Cav-1a expression with the MO, fluorescently labeled caveolae were not observed.

### FPALM Imaging *in vivo* Enables Penetration into Zebrafish with Sub-Diffraction Limited Resolution

Although single molecule microscopy in a living organism has been demonstrated with TIRF [29], TIRF does not enable imaging of thick samples because its setup restricts the excitation of fluorophores to those in close proximity to the coverglass (~100 nm thickness) [30]. This optical section thickness is approximately one-tenth that afforded by confocal fluorescence microscopy techniques. However, confocal microscopy does not approach the resolution levels that super-resolution techniques can provide. Here, FPALM imaging in a zebrafish embryo is demonstrated using widefield illumination, which excites fluorophores in the entire column of laser illumination and thus enables imaging of single molecules in a thick sample. An FPALM image with simple widefield illumination produces a two-dimensional projection of molecules within ~400 nm in the axial direction, for the numerical aperture (NA) of the objective lens used here, as determined by the axial focal volume of the PSF. Excitation of molecules over a wide range of axial positions with widefield illumination in principle allows three-dimensional imaging with biplane [6] or astigmatism [7] to be performed *in vivo*.

**Figure 4:**
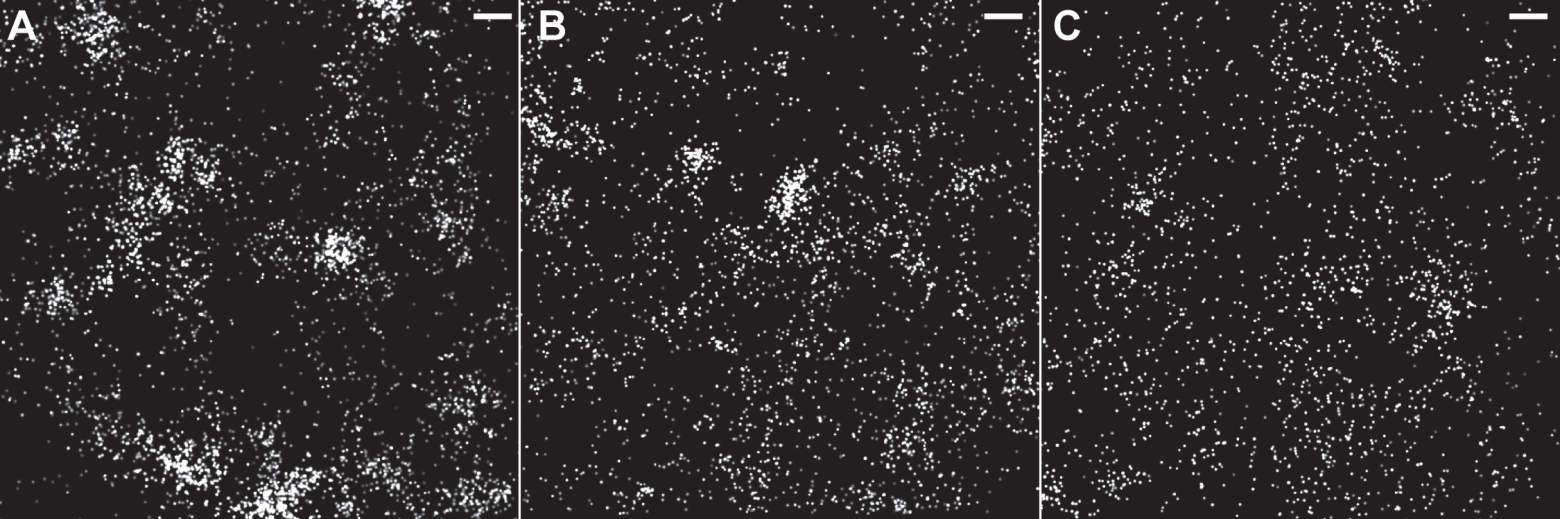
Widefield FPALM Enables Penetration Into Sample. Single molecule localization with FPALM has greater Z-depth into the sample than TIRF microscopy. Images show a region of a zebrafish cell after localization of single molecules. *Casper fms* zebrafish were injected at the 1-cell stage with plasmid DNA and images were captured 24 h post injection (i.e. 24 hpf). *(A)* Image at focal plane of fish with high dendra2 expression (0 μm/10,046 molecules); *(B)* Image shown 5 μm above the focal plane depicted in A (9319 molecules); *(C)* Image shown 5 μm above the focal plane depicted in B, or 10 μm above the focal plane depicted in A (4434 molecules). *Scale bar*, *500 nm*.

Zebrafish embryos were injected at the 1-cell stage with both plasmid DNA (pcDNA3.1 cav1a-HL4 dendra2) and manually dechorionated at 24 h post injection, prior to imaging. Embryos were immersed in tricaine and imaged as shown in Fig. 1. The focal plane for imaging was determined based on observations of 405-nm-dependent single molecule photoactivation and a high density of molecules visible within each camera frame. Regions of interest from ten cells each from two different fish were analyzed (n = two separate experiments). Data for rendered images was captured in this plane (Fig. 4*A*). After acquisitions within this plane were completed, the imaging plane was moved up (deeper into the embryo) by ~5 μm for imaging, and a new data set of 5000 frames was acquired (Fig. 4*B*). This axial translation was repeated once more such that a third acquisition was obtained with the imaging plane ~10 μm into the sample, relative to the initial imaging plane (Fig. 4*C*) (for a total of three acquisitions spaced evenly ~5 μm apart over a total range of 10 μm). At the original focal plane, 10,046 molecules were localized. Images taken deeper into the sample had fewer molecules (9,319 and 4,434 molecules, respectively). Thus, unlike TIRF microscopy, the ability to move the focal plane, combined with widefield illumination for FPALM, yielded large numbers of molecules acquired at different depths, allowing possibilities of section-specific studies of living tissues *in vivo*.

### Caveolae-like Structures Visible at Cell Membrane

**Figure 5:**
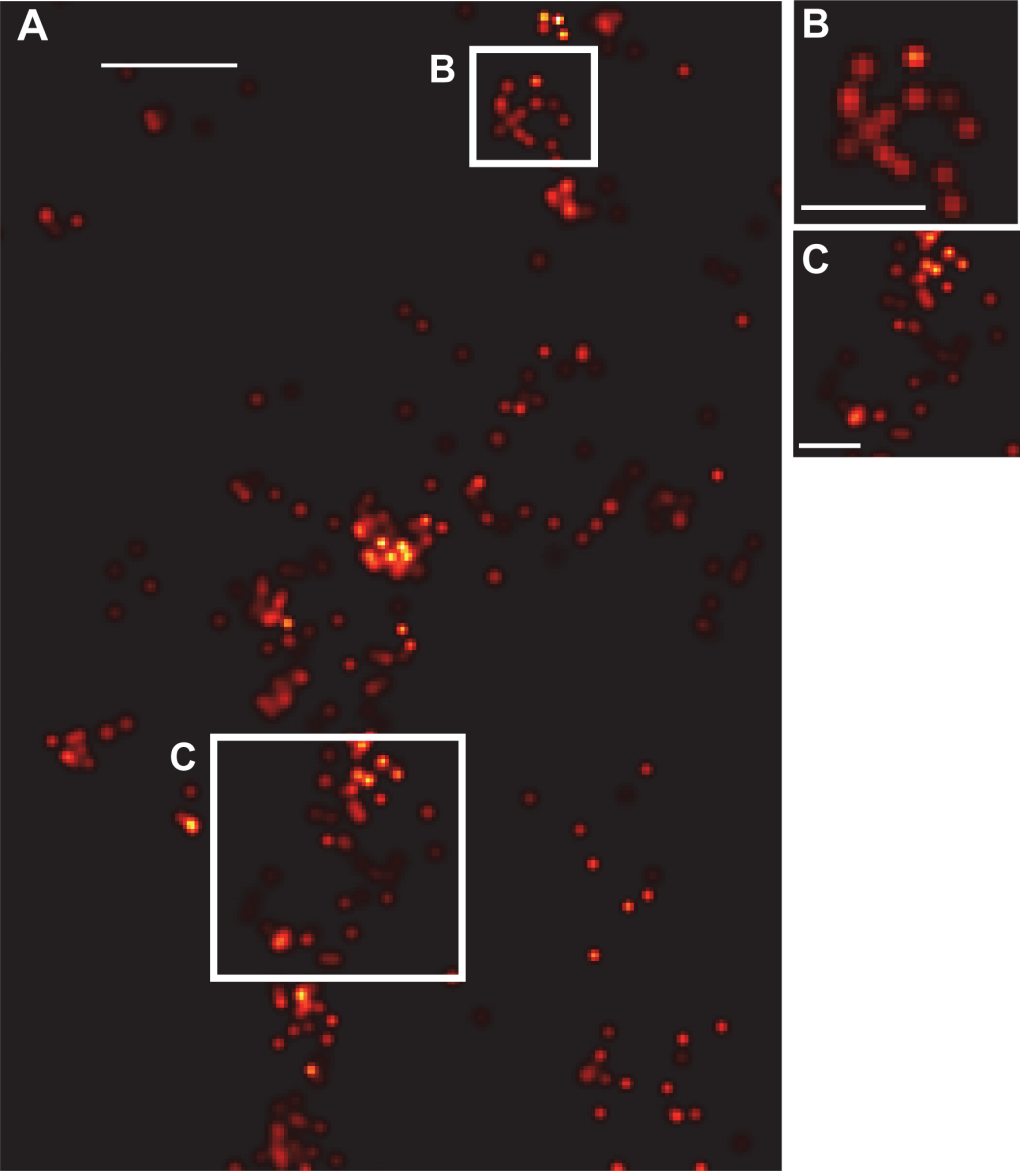
Caveolae-like structures evident at cell membrane. (A) Shown are molecules along the membrane of one cell representative of the experiment, where clusters of Cav-1 molecules can be seen. Magnifications (B, C) of the regions marked by the white boxes in A show structures indicative of caveolae. *Scale bar for A*, *250 nm*. *Scale bar for B-C*, *125 nm*.

An important question was whether we were definitively imaging caveolae, and whether, after overexpression of Cav-1a, imaged structures represented native caveolae. Fig. 3 demonstrated the specificity with which we were imaging Cav-1a. However, in order to look more closely at the structure being imaged, we performed additional experiments looking at the cell membrane for evidence of caveolae-like morphology (Fig. 5). We assessed the size of caveolae-like clusters and counted the number of molecules per cluster in membrane clusters that we identified as putative caveolae. Fifteen cells/clusters from two different fish were analyzed from two separate experiments. The average diameter of the observed caveolae was ~124 nm± 7.4, while the number of molecules per caveolae was 116 with a standard error of the mean ± 17. Estimating the density of localizations for a circular domain of 124 nm diameter, we obtain an area of *A* = πr^2^ = 0.0123 μm^2^, yielding a density of ~9500 molecules/μm^2^, or an average nearest neighbor distance of *d* = ~10.3±0.8 nm.

## Discussion

In this study, we performed super-resolution microscopy in a living zebrafish embryo. By studying living zebrafish embryos, we extended FPALM to *in vivo* imaging of a vertebrate organism. Performing FPALM in a living zebrafish embryo is a major extension of super-resolution microscopy and opens the possibility for nanoscale imaging of proteins (and other molecules) of interest in a living organism. *In vivo* FPALM exploits the optical clarity of the zebrafish to image biological structures beyond the diffraction limit with resolutions limited only by density and localization precisions of molecules. Such studies can yield a greater level of understanding of caveolae domains. Caveolae are important for signal transduction, are utilized by pathogens to gain entry into host cells, and are important in organizing antiviral receptor molecules for host innate immune response [14].

Recent publications have reported the use of stimulated emission depletion (STED) to image *Caenorhabditis elegans* (*C*. *elegans*) nematodes expressing a GFP-fusion protein to look at neurons with an approximate resolution of <60 nm [11], PALM to image an actin protein in live bacteria cells at ~40 nm resolution [12,31], and TIRF to visualize molecules and perform single particle tracking in a zebrafish cell line and also in 2-day-old zebrafish embryos [29]. From our results we estimate a localization precision of *σ*_xy_ = ~40 nm. Because the nearest neighbor distance *d* = ~10.3±0.8 nm is much smaller than *σ*_xy_, the resolution is limited primarily by *σ*_xy_, and therefore can be estimated as $r∼\sqrt{\sigma_{xy}^{2}+{\left(2d\right)}^{2}}=45\;\mathrm{nm}$. [26,32]. This is consistent with the size of caveolae we measured (124 nm ± 7.4 nm), which is expected to be somewhat larger than the actual size (50–80nm) due to the (45 nm) resolution *in vivo*. Similarly to zebrafish, *C*. *elegans* are transparent and small in size, and therefore amenable to microscopy studies of biological functions *in vivo*. Single molecule investigations such as these have led to important insights into intracellular processes nematodes or bacteria. However, these models greatly lack the complexity and genetic similarity to humans needed in an advanced animal model for many aspects of human health and TIRF is limited to imaging a thin layer in close proximity to the coverslip. Our research interests led us to seek a method for extending the field of super-resolution microscopy to studies within a vertebrate organism displaying functional similarities to humans.

We selected cav-1, the primary protein responsible for the formation of caveolae domains [17,18] for these FPALM studies due to the distinct clustering of the protein and unique shape, combined with the fact that we had performed previous studies studying cav-1 in zebrafish cell culture [14]. Forming a characteristic flask or Ω-shape in three dimensions, these caveolae domains appear as a dense cluster of molecules in two dimensional images of the membrane, as shown in Figs. 3–5 and previous studies in cell culture [14]. While the size of the observed caveolae *in vivo* (124 nm ± 7.4) is larger than published reports from electron microscopy studies of 50–80 nm, this may be attributed to the finite size of the localization precisions, which tend to form convolutions of the actual structure to yield slightly larger and blurred images, as in all localization microscopy methods. The number of molecules/cluster (~116; s.e.m. is ± 17) is comparable to that which we have previously observed *in vitro* (~135 molecules/cluster) [14]. Due to their sub-diffraction size, caveolae have been difficult to image using diffraction limited fluorescence microscopy, however FPALM affords the ability to image caveolae in a living organism. Both caveolae and the main protein components of caveolae, the caveolins, have been linked to a variety of human diseases such as cancer, diabetes, atherosclerosis, Alzheimer’s, and muscular dystrophy [17]. Here, we have demonstrated the ability to image caveolae in a living zebrafish. Further, we have shown the ability to localize molecules up to 10 μm within the live zebrafish, but believe that deeper penetration is possible, within the range afforded by the working distance of the objective lens, under controlled amounts of fluorescence scattering and background. Current studies are underway to determine the fundamental limitations on depth.

The imaging parameters discussed herein are not exhaustive, and current work is being performed to provide additional options and improvements to the FPALM imaging described. A primary concern includes locating proteins of interest within the zebrafish or knowing which cells are being imaged. In contrast to cell culture experiments where monolayer cells are being imaged or TIRF [29] imaging with a limited sample penetration depth (~100 nm thickness), we are imaging into a thick, three dimensional sample. Such problems can be alleviated by tissue-specific labeling techniques and locating fluorescence signals from the labels, which do not overlap with fluorescence emission used for FPALM imaging. Due to the shape of the cells imaged *in vivo*, we predict that we are looking at epithelial or fibroblast cells. The studies described here were performed using a protein of interest that was structurally distinct and that has also been imaged *in vitro* [14]. Embryos were first examined for expression of unconverted Dendra2 and scanned until images on the camera screen showed flashes of light consistent in size and duration with PA-FPs (~2–3 pixels for ~ 10 ms over 2–4 frames) being activated and showing single molecule fluorescence. One advantage of using this construct was that Cav-1 molecules are highly expressed, which further facilitated imaging with greater penetration into the embryo, where protein density was still sufficient to image large numbers (thousands) of single molecules to provide sufficient density of molecules and thus high image resolutions. Labeling density is an important consideration because a sufficient number of localizations are needed to define a structure with enough resolution, and the number of localizations is limited by the density of labels on the protein of interest.

Providing a noninvasive tool for super-resolution imaging, *in vivo* FPALM can enable investigations of many more types of intracellular processes. The present study demonstrates the possibility of extending *in vivo* single molecule super-resolution imaging to a wide variety of new applications. Transgenic zebrafish lines that stably express photoactivatable fluorescent fusion proteins could be used, or new ones generated, in a *casper fms* genetic background so that investigations can be performed at later developmental stages of the zebrafish. Utilizing a photoconvertible probe such as dendra2 that enables pre-screening of embryos with fluorescence from its inactive form is recommended, since the photophysical properties of the probe used will play a significant role in the spatial resolution and the rate of image acquisition. The zebrafish is an ideal model organism for a variety of processes such as infection and immunity [33–42], cancer [40,41,43], and muscular dystrophy [44,45] and *in vivo* FPALM provides the opportunity to provide insights into mechanisms underlying a variety disease processes at the molecular level.

Critical considerations are the motion of the sample during the acquisition and background noise. It is important to consider how the motion of individual proteins within the structure of interest may affect localization precision, and hence, image resolution. In our studies, we observed lateral drift of ~60–70 nm over the image acquisition time of ~3 minutes. However, we also demonstrated here the ability to use cross-correlation and perform post-acquisition drift correction using transmitted light images taken before and after image acquisition. Drift correction is an important step in the image analysis of super-resolution imaging, as sample motion or drift on the order of the localization precision may lead to misinterpretation of data. Background noise becomes more pronounced when imaging inner sections of a thick sample. In our studies, typical background noise observed was ~7–12 photons. The use of the *casper fms* transgenic zebrafish line greatly alleviated background due to the lack of melanocytes and iridophores in the *casper fms* embryos [19]; however, the fish still featured measurable levels of inherent autofluorescence and out-of-focus background. Probe choice is again important when considering background, as using probes with large contrast will improve signal-to-background ratios. Additionally, probes with longer wavelength emission may help minimize detected background from having reduced light scattering.

Still, further possibilities for exploiting the optical transparency of zebrafish for FPALM imaging are numerous and offer many advantages over previously described techniques. In principle, *in vivo* imaging with FPALM is compatible with polarization-FPALM (P-FPALM) [46], biplane-FPALM (BP-FPALM) [6] and multicolor imaging [9]. To achieve combinations of these techniques would require the addition of a polarizing beamsplitter, a 50:50 beamsplitter or a dichroic mirror, as well as mirrors, and lenses to the original FPALM detection path. Similar to the multi-color imaging *in vitro* previously demonstrated [9], employing additional PA-FPs for *in vivo* colocalization imaging experiments can be easily achieved. Optimal probe combinations with spectral separation, high brightness, contrast and rate of photon emission should be used to further progress toward nanoscale colocalization of proteins of living vertebrate organisms *in vivo*.

The advent of super resolution microscopy imaging techniques such as FPALM is pushing the biomedical sciences into an era where imaging below the diffraction limit is becoming as commonplace as conventional imaging currently has been in the past. We have demonstrated a method for imaging single molecules in a living organism with resolution below the diffraction limit. The imaging capabilities and possibilities of *in vivo* FPALM should substantially benefit investigations into biological functions in a living vertebrate organism with relevance to human diseases. Applications of *in vivo* FPALM techniques provide the opportunity to ask and answer a multitude of questions in a living organism with diffraction-unlimited resolutions. New biological insights are hopefully imminent.
